# ICD-11 Prolonged Grief Disorder Criteria: Turning Challenges Into Opportunities With Multiverse Analyses

**DOI:** 10.3389/fpsyt.2020.00752

**Published:** 2020-08-07

**Authors:** Maarten C. Eisma, Rita Rosner, Hannah Comtesse

**Affiliations:** ^1^Department of Clinical Psychology and Experimental Psychopathology, University of Groningen, Groningen, Netherlands; ^2^Department of Psychology, Catholic University Eichstaett-Ingolstadt, Ingolstadt, Germany

**Keywords:** prolonged grief disorder, persistent complex bereavement disorder, complicated grief, criteria, algorithm, criticism, critique, multiverse analyses

## Introduction

Recently, prolonged grief disorder (PGD), a diagnosis characterized by severe, persistent and disabling grief, was formally included in the 11th revision of the International Classification of Diseases [ICD-11; (1): **Table 1**]. To meet PGD_ICD-11_ criteria one needs to experience persistent and pervasive longing for the deceased and/or persistent and pervasive cognitive preoccupation with the deceased, combined with *any* of 10 additional grief reactions assumed indicative of intense emotional pain for at least six months after bereavement. Contrary to the 5th revision of the Diagnostical and Statistical Manual of Mental Disorders [DSM-5; (11)] and the 10th revision of the International Classification of Diseases [ICD-10; (12)], the ICD-11 only uses a typological approach, implying that diagnosis descriptions are simple and there is no strict requirement for the number of symptoms one needs to experience to meet the diagnostic threshold.

**Table 1:** Overview of ICD-11 diagnostic criteria for PGD and results of multiverse analyses of PGD_ICD-11_.

ICD-11 criteria for PGD	Study	Sample	Measure ^1^	Results
A. At least one of the following: a persistent and pervasive longing for the deceased or a persistent and pervasive preoccupation with the deceased.	Boelen and Lenferink (2)	855 bereaved community members	ICG-R, SCL-90 depression scale	**Prevalence rate of PGD_ICD-11_ and diagnostic agreement with PCBD, PGD_2009,_ PGD_ICD-11 BD_, CG, and PGD_DSM-5_**^2^**, respectively, for various additional symptom thresholds:** 1+ = 19.8%, κ = .66,.67,.98,.92,.93; 2+ = 18.1%, κ = .69,.73,.96,.89,.94; 3+ = 16.1%, κ = .75,.79,.89,.82,.89; 4+ = 13.4%, κ = .78,.82,.79,.72,.81; 5+ = 10.8%, κ = .83,.82,.67,.61,.72; 6+ = 7.8%, κ = .76,.76,.52,.47,.56; 7+ = 5.0%, κ = .59,.57,.36,.32,.39 **Number of possible symptom combinations to meet PGD_ICD-11_ caseness for various additional symptom thresholds**: 1+ = 3069, 2+ = 3039, 3+ = 2904, 4+ = 2544, 5+ = 1914, 6+ = 1158, 7+ = 528
B. Examples of intense emotional pain Accompanied by intense emotional pain e.g. sadness, guilt, anger, denial, blame; difficulty accepting the death; feeling one has lost a part of one’s self; an inability to experience positive mood; emotional numbness; difficulty in engaging with social or other activities.	Boelen et al. (3)	551 bereaved community members	ICG-R, SCL-90 depression scale	**Prevalence rate of PGD_ICD-11_ and diagnostic agreement with PCBD for various additional symptom thresholds:** 1+ = 19.2%, κ = .51, 2+ = 17.6%, κ = .56; 3+ = 15.4%, κ = .62; 4+ = 11.1%, κ = .75; 5+ = 8.3%, κ = .84, 6+ = 5.3%, κ = .71
	Bonanno and Malgaroli (4)	282 bereaved community members	“Structured clinical interview”	**Prevalence rate of PGD_ICD-11_ and diagnostic agreement with PCBD for various symptom thresholds:** 1+ = 11.7%, percentage match = 36.4%, 3+ = 8.8%, percentage match = 48.0%; 5+ = 4.6%, percentage match = 76.9%
C. Time and impairment Persisted for an abnormally long period of time (more than 6 months at a minimum): following the loss, clearly exceeding expected social, cultural or religious norms for the individual’s culture and context.	Comtesse et al. (5)	113 bereaved treatment-seekers	ICG, PG-13	**Sensitivity and specificity values from ROC analysis of PGD_ICD-11_ for various additional symptoms thresholds (interview-diagnosed PGD_2009_ as criterion standard):** 1+ = 100% and 6%, 2+ = 100% and 9%, 3+ = 100% and 30%, 4+ = 98% and 56%, 5+ = 88% and 75%, 6+ = 82% and 92%, 7+ = 65% and 96%, 8+ = 37% and 100%, 9+ = 15% and 100%, 10+ = 7% and 100% **Prevalence rate of PGD_ICD-11_ and diagnostic agreement with PCBD for various additional symptom thresholds**: 1+ = 69.1%, κ = .51; 6+ = 46.9%, κ = .77
The disturbance causes significant impairment in personal, family, social, educational, occupational or other important areas of functioning.	Cozza et al. (6)	1732 bereaved military family members	CGQ	**Diagnostic agreement of PGD_ICD-11_ with PCBD, PGD_2009,_ and CG, respectively, for thresholds of one or two additional symptoms for all criteria-sets:** κ for PGD_ICD-11_ and PCBD = .90 or.87, κ for PGD_ICD-11_ and PGD_2009_ = .89 or.86, κ for PGD_ICD-11_ and CG = .93 or.89
	Mauro et al. (7)	261 bereaved treatment-seekers	SCI-CG	**Diagnostic agreement of PGD_ICD-11_ with ICG cut-off ≥ 30 and clinician-rated CG and functional impairment for various additional symptom thresholds**: 0+ = 95.8%, 1+ = 95.8%, 2+ = 95.0%, 3+ = 91.6%, 4+ = 86.2%, 5+ = 72.8%, 6+ = 45.2% (5+ and 6+ estimates fall in between PGD_2009_ rates of agreement: 59.0%)

PGD, prolonged grief disorder; PGD_ICD-11_, ICD-11 diagnostic criteria for PGD; PGD_ICD-11 BD_, diagnostic criteria according to the ICD-11 beta draft [or a description see (2)]; PGD_2009_, diagnostic criteria for PGD according to Prigerson et al. (8); PCBD, DSM-5 diagnostic criteria for persistent complex bereavement disorder; CG, diagnostic criteria for complicated grief (9); CGQ, Complicated Grief Questionnaire; ICG, Inventory of Complicated Grief; ICG-R, Inventory of Complicated Grief Revised; PG-13, Interview for Prolonged Grief-13; SCI-CG, Structured Clinical Interview for Complicated Grief; SCL-90, Symptom-Checklist-90. ^1^ In all cited studies, items of the respective measure(s) were used to assess (approximations of) PGD_ICD-11_ symptoms. Symptoms were regarded as present if judged as ‘present’ by an interviewer (4) or if an item was scored higher than a specific value (e.g., ≥ 4) on the respective scale(s) (e.g., five-point Likert scale). Each symptom score was dichotomously coded as “absent” (0) or “present” (1). PGD_ICD-11_ caseness was then determined following the ICD-11 diagnostic rule. ^2^ PGD_DSM-5_ was interpreted here according to proposed guidelines [(10), i.e., three additional symptoms].

Some researchers have argued that PGD_ICD-11_’s typological approach is helpful, as it will lead to greater sensitivity in case identification in clinical practice and increased cross-cultural applicability (13). Others have highlighted that the typological approach allows for flexible diagnostic algorithms in research, so that PGD_ICD-11_ criteria can be adapted to resemble the characteristics of both stricter and more lenient precursor criteria (14). In the current contribution, we take a different, complementary position. We highlight a series of challenges in using the PGD criteria for research purposes and discuss the application of a method that employs flexibility of PGD_ICD-11_ diagnostic approach to address these challenges, which may help in working toward the unbiased, structured, and transparent identification of optimal criteria for disturbed grief.

## A Critique of PGD ICD-11 for Research Purposes

A first challenge to researchers applying PGD_ICD-11_ criteria is that they were completely new when first introduced and differed substantially from previously proposed diagnostic criteria sets (15). For example, PGD_ICD-11_ contains multiple symptoms not found in any prior proposed criteria set, such as guilt, blame and the inability to experience positive mood [for a full criteria set comparison: (16)]. Furthermore, oft-used measures to assess disturbed grief responses, such as versions of the Inventory of Complicated Grief [e.g., ICG; (17)] do not fully assess PGD_ICD-11_ criteria [6; for a recent review illustrating this point: (18)]. Therefore, the development of new, reliable and valid instruments is critical to assess the characteristics and validity of PGD_ICD-11_ and determine for common research purposes [e.g., establishing prevalence, risk factors, treatment efficacy] who meets PGD_ICD-11_ criteria. However, the typological approach in PGD_ICD-11_ poses a substantial challenge to the development of such instruments.

First, the plain language used to formulate additional criteria makes it unclear what precisely each criterion implies. Single-word criteria “guilt,” “anger,” “denial,” and “blame” are particularly problematic. For example, “blame” could refer to self-blame or other-blame, blame for the death, or blame for something else. Since self-blame is much more prevalent in bereaved persons than blaming others (19) the interpretation of this criterion influences its prevalence. Moreover, blaming oneself for the death perpetuates disturbed grief, whereas blaming others for the death does not (20), so the characteristics and clinical correlates of this criterion may be very different depending on how it is interpreted [for a related discussion: (21)].

Second, even core criteria of “longing” and “preoccupation,” shared with most prior proposed grief disorders, are potentially problematic in their implementation. For example, it is unclear if a higher item-score threshold should be used to indicate that someone experiences persistent and pervasive longing, as it is the most frequently reported experience in bereavement (22), and, as a consequence, one of the least sensitive criteria in distinguishing those with and without disturbed grief (14). Preoccupation with the deceased is also already being interpreted differently by influential researchers in the grief field, with some viewing it as intrusive images about the death (23), and others as a process similar to grief rumination (24). While imagery and rumination are related processes, they are dissimilar in phenomenal characteristics, such as their duration, sensory experiences, and emotional correlates (25). The interpretation of this criterion will therefore have repercussions for what we regard as PGD.

Third, many key characteristics of PGD_ICD-11_ (e.g., prevalence, classification, symptom heterogeneity) depend heavily on the chosen diagnostic algorithm. Pioneering research on characteristics of this disorder (with measures approximating actual criteria) used the minimal criteria as specified in the ICD-11 (i.e., at least one core criterion, and at least one additional criterion) in addition to the time and disability criteria (7, 26). It soon became apparent that applying these minimal criteria led to much higher prevalence rates for PGD_ICD-11_ than for prior proposed criteria of PGD [PGD_2009_; (8)] and persistent complex bereavement disorder [PCBD; DSM-5, (10)]. This algorithm is thus relatively lenient, and applying it may lead to overdiagnosis and limited generalizability of findings on two of the most-studied grief disorder proposals (i.e., PGD_2009_; PCBD) to PGD_ICD-11_ (14). This elicits the question: If the diagnostic algorithm directly derived from the ICD-11 text is too liberal, which diagnostic rules are then optimal for research?

## Multiverse Analyses in Research on PGD ICD-11

In summary, a fundamental challenge for grief researchers in using the PGD_ICD-11_ diagnosis is that its criteria are open for multiple interpretations and that the only diagnostic algorithm mapping one-on-one on the diagnosis description is too lenient. While the current criteria cannot easily be amended, their systematic investigation can make them more useful to researchers, for instance by providing a basis for achieving consensus on symptom interpretation, algorithms, and future PGD criteria. We propose that multiverse analyses can be particularly helpful in achieving such goals. Multiverse analyses typically consist of a procedure wherein one performs similar analyses across multiple datasets generated by making reasonable but variable choices on excluding, transforming and coding data (27). For example, when using a reaction time task with skewed data, one may perform analyses based on the median or the mean or analyze the data using parametric or non-parametric statistical tests. By comparing outcomes of multiple analyses, one can establish the degree of uncertainty about the conclusions one arrives at and the robustness of findings to arbitrary decisions made in data preparation and analysis. For example, one may discover that the direction and significance of effects is similar regardless of these decisions or that some decisions lead to significant effects, whereas others do not. The first scenario would allow for strong conclusions and the second scenario would signal caution is warranted in the interpretation of findings.

We advocate a similar but conceptually distinct procedure wherein empirical research examining the characteristics of PGD_ICD-11_ systematically vary certain aspects of these criteria (e.g., using a more stringent cut-off for longing) or the diagnostic algorithm (e.g., varying the number of additional symptoms). A comparison of results obtained with multiple interpretations of criteria can help illuminate how robust specific results are dependent on multiple interpretations of the PGD_ICD-11_ criteria. For example, one may be able to investigate the robustness of group differences between people with and without PGD on risk-factors and protective factors or treatment effectiveness (e.g., percentage with and without diagnosis after treatment) dependent on different interpretations of PGD_ICD-11_. Additionally, critical information can be gathered on the influence of variations in symptom interpretations and algorithms on PGD_ICD-11_ characteristics and how these characteristics compare to other proposed criteria sets [e.g., the newly developed PGD criteria for the upcoming text revision of DSM-5, (10)]. That is, multiverse analyses can be applied to shed light on a variety of clinically relevant characteristics of PGD_ICD-11_ (e.g., retest and interrater reliability, specificity and sensitivity of classification, distinctiveness from other disorders, associations with functional impairment) when systematically modifying interpretations of its criteria.

Only a handful of studies have thus far applied such analyses, which have predominantly narrowly focused on examining characteristics of PGD_ICD-11_ and their comparability against external standards when varying the number of additional criteria (see **Table 1** for a summary). It has been observed that minimal PGD_ICD-11_ criteria yield a similar prevalence as the relatively lenient Shear et al. criteria (9) for complicated grief (7), but almost two times higher prevalence than relatively strict PCBD criteria (2, 28, 29). Multiverse analyses in community samples demonstrated that similar prevalence estimates and good diagnostic agreement with PCBD and PGD_2009_ appears to be achieved with five additional criteria for PGD_ICD-11_ [(2–4), cf. (7)]. Similarly, in treatment-seekers, minimal PGD_ICD-11_ criteria correctly classified people against a relatively lenient standard of 30 or higher on the ICG (6, 7), yet as many as six additional symptoms were necessary to yield comparable prevalence and good diagnostic agreement with PGD_2009_ and PCBD (5). Moreover, one study demonstrated the influence of the number of additional symptoms on symptom heterogeneity, theoretically demonstrating that the number of ways to meet PGD_ICD-11_ criteria ranges from 3.069 for one to 528 for seven additional symptoms (2). A less heterogeneous diagnosis is clearly preferable, as it would lead to less variability within groups of people meeting grief disorder criteria, making the distinction between these people more useful for research and practice (30). Taken together, these examples illustrate that the properties of PGD_ICD-11_ depend on both the chosen diagnostic rule and the stringency of the comparison standard, and that the number of additional symptoms is critical in determining prevalence, clinical classification, and symptom profiles.

For future research, we recommend multiverse analyses varying not only the algorithm, but additionally symptom interpretations of single-item criteria and cognitive preoccupation and cut-offs for the presence of the longing criterion. We also advise to substantially expand the current focus of multiverse analyses of PGD_ICD-11_ to establish the robustness of clinically-relevant findings (e.g., on treatment efficacy) and the variety of other aspects relevant to the validity of a diagnosis [for reviews: (13, 24)]. The latter includes - but is not limited to: reliability of classification [e.g., (31)], the structure of symptoms [e.g., (32)], distinctiveness from related disorders [e.g., (33)], and relationships with functional impairment [e.g., (34)]. We further advocate transparency in applying multiverse analyses and recommend: open access publication, data accessibility (e.g., through availability in repositories), fully specifying the origins and formulations of items used to assess PGD_ICD-11_ and, if applicable, other disturbed grief criteria, and complete reporting of the variations of PGD_ICD-11_ and outcomes under investigation.

## Discussion

In the absence of clearly defined criteria and diagnostic rules for PGD_ICD-11_, researchers should broadly apply structured methods to examine the characteristics of this disorder and compare it against past and future proposed grief disorders. Multiverse analyses can be a powerful tool to determine the validity and clinical usefulness of the PGD_ICD-11_ criteria. By systematically varying the number of core and additional symptoms, the interpretation of symptoms, and the standards for meeting symptoms, we can evaluate how such decisions influence the characteristics of PGD_ICD-11_, also in relation to different external standards. This will create a comprehensive research base enabling us to enhance our understanding of PGD_ICD-11_ and of disturbed grief more generally. Creating this research base is no panacea: it cannot undo the inherent weaknesses of the PGD_ICD-11_ criteria. However, the systematic evaluation of this information will help clarify under which circumstances diagnoses behave similarly or differently, providing a stepping stone to harmonize PGD_ICD-11_ criteria with other criteria sets and to develop more optimal future disturbed grief criteria.

## Author Contributions

All authors developed the ideas for this article. ME and HC wrote the first draft of the manuscript. RR contributed important intellectual content by providing critical revisions to the first draft.

## Funding

ME was supported by a Netherlands Organization for Scientific Research (NWO) Veni grant [Grant ID: 016.veni195.113]. The funder did not play a role in the study design, the writing of the report or in the decision to submit the article for publication. RR was supported by a grant of Deutsche Forschungsgemeinschaft [Grant ID: RO2042/7-1]. The funder did not play a role in the study design, the writing of the report or the decision to submit the article for publication.

## Conflict of Interest

The authors declare that the research was conducted in the absence of any commercial or financial relationships that could be construed as a potential conflict of interest.
